# Optimization of the Chronic Kidney Disease–Peritoneal Dialysis App to Improve Care for Patients on Peritoneal Dialysis in Northeast Thailand: User-Centered Design Study

**DOI:** 10.2196/37291

**Published:** 2022-07-06

**Authors:** Eakalak Lukkanalikitkul, Sawinee Kongpetch, Wijittra Chotmongkol, Michael G Morley, Sirirat Anutrakulchai, Chavis Srichan, Bandit Thinkhamrop, Theenatchar Chunghom, Pongsai Wiangnon, Wilaiphorn Thinkhamrop, Katharine E Morley

**Affiliations:** 1 Center of Excellence in Kidney Diseases, Srinagarind Hospital Faculty of Medicine Khon Kaen University Khon Kaen Thailand; 2 Ophthalmic Consultants of Boston Harvard Medical School Boston, MA United States; 3 Division of Nephrology, Faculty of Medicine Khon Kaen University Khon Kaen Thailand; 4 Department of Computer Engineering, Faculty of Engineering Khon Kaen University Khon Kaen Thailand; 5 Data Management and Statistical Analysis Center Faculty of Public Health Khon Kaen University Khon Kaen Thailand; 6 Kidney Unit, Srinagarind Hospital Faculty of Medicine Khon Kaen University Khon Kaen Thailand; 7 Massachusetts General Hospital Center for Global Health Harvard Medical School Boston, MA United States

**Keywords:** peritoneal, dialysis, peritoneum, mobile health, mHealth, rapid cycle process improvement methodology, home monitoring, near-field communication, monitor, kidney, rapid cycle improvement, quality improvement, process improvement, methodology, nephrology, nephrologist, internal medicine, computer program, Unified Theory of Acceptance and Use of Technology, UTAUT, usability, interface, metric capture, barrier, renal, mobile phone

## Abstract

**Background:**

The prevalence of peritoneal dialysis (PD) in Thailand is increasing rapidly in part because of Thailand’s *Peritoneal Dialysis First* policy. PD is a home-based renal replacement therapy in which patients with chronic kidney disease perform up to 4 exchanges of dialysate fluid per day in the peritoneal cavity. Overhydration is one of the most common complications in patients on PD and is associated with increased morbidity and mortality. To monitor hydration status, patients collect hydration metrics, including body weight, blood pressure, urine output, and ultrafiltration volume, from each dialysis cycle and enter this information into a PD logbook. This information is reviewed bimonthly at PD clinic appointments. The chronic kidney disease-PD (CKD-PD) app with near-field communication (NFC) and optical character recognition (OCR) was developed to automate hydration metric collection. The information was displayed in the app for self-monitoring and uploaded to a database for real-time monitoring by the PD clinic staff. Early detection and treatment of overhydration could potentially reduce the morbidity and mortality related to overhydration.

**Objective:**

This study aims to identify usability issues and technology adoption barriers for the CKD-PD app with NFC and OCR and a monitoring system and to use this information to make rapid cycle improvements.

**Methods:**

A multidisciplinary team of nephrologists, PD clinic nurses, computer programmers, and engineers trained and observed 2 groups of 5 participants in the use of the CKD-PD app with NFC and OCR and a monitoring system. The participants were observed using technology in their homes in 3 phases. The data collected included the Unified Theory of Acceptance and Use of Technology questionnaire, *think-aloud* observation, user ratings, completion of hydration metrics, and upload of hydration metrics to the central database. These results were used by the team between phases to improve the functionality and usefulness of the app.

**Results:**

The CKD-PD app with NFC and OCR and a monitoring system underwent 3 rapid improvement cycles. Issues were identified regarding the usability of the NFC and OCR data collection, app stability, user interface, hydration metric calculation, and display. NFC and OCR improved hydration metric capture; however, issues remained with their usability. App stability and user interface issues were corrected, and hydration metrics were successfully uploaded by the end of phase 3. Participants’ scores on technology adoption decreased but were still high, and there was enthusiasm for the self-monitoring and clinical communication features.

**Conclusions:**

Our rapid cycle process improvement methodology identified and resolved key barriers and usability issues for the CKD-PD app with NFC and OCR and a monitoring system. We believe that this methodology can be accomplished with limited training in data collection, statistical analysis, and funding.

## Introduction

### Chronic Kidney Disease and Peritoneal Dialysis in Northeast Thailand

Chronic kidney disease (CKD) is a major health problem in Thailand because of its high prevalence, cost of treatment, significant morbidity and mortality, and substantial impact on the quality of life of patients and their families. From 2017 to 2018, the Chronic Kidney Disease Prevention in the Northeast of Thailand (CKDNET) project found that the prevalence of CKD was 27% in the rural provinces of northeast Thailand [1], primarily because of rising rates of diabetes, hypertension, and other primary renal diseases [2]. Despite this increased need, there is only 1 nephrologist for every 593,000 population compared with every 44,000 population in Bangkok [3]. In 2008, the Thai government adopted the *Peritoneal Dialysis* *First* policy for renal replacement therapy under its universal health care coverage scheme, increasing access in low-resource settings, with approximately 21% of patients on peritoneal dialysis (PD) in northeast Thailand [4]. Patients on PD manage their PD at home manually or by using a PD cycler to deliver dialysate fluid through a catheter placed in the peritoneal cavity, where the fluid remains for several hours. They enter hydration metric data, including body weight, blood pressure, urine output, and ultrafiltration volume for each cycle, in handwritten notebooks for review by a nephrologist at bimonthly clinic appointments.

### User Design and Evaluation of Successful Adoption of the CKD-PD App

In 2018, the Data Management and Statistical Analysis Center, Faculty of Public Health, Khon Kaen University, developed the CKD-PD mobile app to help nephrologists and patients on PD manage fluid status to prevent overhydration. It is a common complication in patients on PD. Overhydration increases morbidity because of PD-related peritonitis, stroke, congestive heart failure, major adverse cardiac events, and mortality [4-9]. The CKD-PD app has been of interest not only to nephrologists but also to the Thai Health Security Office as an intervention for improving the care of patients on PD. Early treatment of overhydration can decrease these related complications, thereby reducing hospitalization and health care expenses [10].

Many apps fail because of a lack of evaluation and removal of barriers to user adoption, fidelity of the technology, and design of the health care delivery system in which the app will be deployed [11,12]. Achieving rapid design and deployment of digital health interventions are 2 challenges facing the successful adoption and implementation of mobile health (mHealth) technologies [13]. This is especially true in low- and middle-income countries where mHealth interventions have been touted as solutions to a wide variety of health care challenges [14-16]; however, little is known about how they perform [17-19]. Before studying the effectiveness of the CKD-PD in managing overhydration in a real-world setting, a user design study was conducted using rapid cycle process improvement methods.

The objectives of this study were to (1) optimize the design and usability of the CKD-PD app and test the app in the context in which it is deployed using rapid cycle process improvement methods; (2) evaluate automatic data entry features using near-field communication (NFC) and optical character recognition (OCR) technology; and (3) identify and address the practical challenges that influence the successful adoption of the CKD-PD app and remote monitoring system in a real-world, low-resource setting.

## Methods

### Study Population

The study was conducted at Srinagarind Hospital, Khon Kaen University, between November 1, 2020, and April 30, 2021. Patients on PD for >3 months without a change in their PD prescription were invited to participate if they met the following inclusion criteria: aged ≥18 years, having access to a smartphone capable of running the CKD-PD app, and willing to allow research staff to observe their use of the CKD-PD app with NFC and OCR features and monitoring equipment in their home. Vulnerable populations specified as pregnant women, children, prisoners, individuals who were institutionalized, or those unable to participate in home data collection were excluded. Informed consent was obtained by the trained research staff. A total of 10 participants were enrolled and divided into group 1 (participants 1-5) and group 2 (participants 6-10). Baseline demographic data, including age, sex, education level, time on PD, and whether they used continuous ambulatory PD (CAPD) or automated PD (APD), were collected at enrollment. Participants were given prepaid cards to cover the cost of study related to internet or cellular data expenses.

### CKD-PD App and Its Features

The CKD-PD app is available for free download in Android and iOS formats and is designed for daily hydration metric collection for patients on PD (Multimedia Appendix 1). Users can enter hydration metrics using manual input or voice recognition. The CKD-PD app graphically displays a patient’s hydration metrics over time and uploads them to the CKDNET database stored in the Thai Care Cloud data repository [20], which is accessible to the PD clinic staff. Nephrologists can set individual hydration parameters for a patient so that they can self-monitor their hydration status. Alerts can be set to notify patients on PD and PD clinic staff when there are actionable hydration metric abnormalities, triggering a prompt review by a nephrologist and allowing the early detection and treatment of overhydration. Another key feature of the CKD-PD app is a direct link using LINE, a social messaging app widely used in Thailand, between the patient and PD clinic, facilitating communication about symptoms and changes in PD management.

A new prototype using NFC and OCR to automate the entry of hydration metric data into the CKD-PD app from measurement devices was developed. NFC uses radio frequency communication and wirelessly transmits data to an NFC-enabled device when it is placed within 4 cm. This offers a simple, low-cost solution that does not require an external power source or pairing, similar to Bluetooth. It is commonly used for mobile payments ad transit cards and, more recently, with subdermal glucose sensors [21-25]. NFC radio frequency tags with unique ID numbers can be added to medical devices such as scales. Users tap a card with an NFC receiver tag on the NFC-equipped scale and then tap it on the NFC card reader, and the data are transferred to the CKD-PD (Multimedia Appendix 2). The CKD-PD app is also equipped with OCR technology, which uses a smartphone camera to capture the digital output from the blood pressure machine and store it in the CKD-PD app.

### Study Design

#### Overview

There were 3 user design phases separated by 2 improvement cycles. During the improvement cycle, the app improvement team analyzed the results from the previous phase and revised the CKD-PD app using the NFC, OCR, and monitoring system (Table 1). The app improvement team included study nephrologists, computer engineers and app developers, and the Data Management and Statistical Analysis Center Thai Care Cloud database team. The app improvement team met as needed to define technology and user design issues, develop solutions, and test modifications.

**Table 1:** Overview and timeline of research activities.

Research activity	Phase 1 (group 1)				IC^a^ 1	Phase 2 (group 2)				IC 2	Phase 3 (groups 1 and 2)	
	Week				Week	Week				Week	Week	
	0	1-2	3	4	5-8	8	9-10	11	12	12-16	17-18	19-20
In-clinic training and observation	✓					✓						
UTAUT^b^ survey	✓			✓		✓			✓		✓	✓
Home observation		✓					✓				✓	✓
Completion of hydration metrics			✓	✓				✓	✓			✓
Contact with PD^c^ clinic			✓	✓				✓	✓			✓
Validation of hydration metrics				✓					✓			✓
Observation logbook											✓	

^a^IC: improvement cycle.

^b^UTAUT: Unified Theory of Acceptance and Use of Technology.

^c^PD: peritoneal dialysis.

#### Phase 1

##### Overview

Group 1 participants received training on the use of the CKD-PD app with the NFC and OCR and monitoring system during week 0. In-home observations were conducted once during weeks 1 to 4, followed by research activities 2 to 3 (see the following sections) during weeks 1 and 2. During weeks 3 and 4, the participants used the CKD-PD app with the NFC and OCR system at their homes and completed research activities 4 to 6 (see in the following sections).

##### Improvement Cycle 1

After completion of phase 1, the results were summarized and presented with the completion of hydration metrics and clinical contact results to the app improvement team. The app improvement team analyzed the results and modified the components of the system, including measurement devices, NFC, OCR, app design, and programming. The user processes were also adjusted.

#### Phase 2

##### Overview

Group 2 participants received training in the use of the CKD-PD app with the NFC and OCR and monitoring system during week 8. In-home observations were conducted during weeks 9 to 12 using the modified CKD-PD app with the NFC and OCR and monitoring system. Research activities 2 to 3 were conducted during weeks 9 and 10, followed by research activities 4 to 6 in weeks 11 and 12.

##### Improvement Cycle 2

After the completion of phase 2, the app improvement team reviewed the new results from participant observations, hydration metric completion, and clinic contacts. Additional issues were identified, and new solutions were developed and tested. Phase 3 was launched after the completion of the improvement cycle 2 modifications.

#### Phase 3

##### Overview

Participants from groups 1 and 2 performed research activities 2 to 6 again during weeks 17 to 20 using the modified CKD-PD app and monitoring system modified without NFC and OCR. This allowed for the evaluation of the usability and functionality of the CKD-PD app without NFC and OCR. In phase 3, participants were asked to rate the entry of body weight and dialysate volumes with NFC again based on their earlier experience for comparison with phases 1 and 2. In addition, research activity 7 (see the following sections) was performed to compare using the usual practice of entering their hydration metrics by hand into a logbook with the CKD-PD app.

##### Final Improvements

After completion of phase 3, the app improvement team made final improvements based on the results of the phase 3 home observation and hydration metric collection using the CKD-PD app and monitoring system without NFC and OCR.

### Description of Study Activities

The details of the study activities are described in the following sections.

#### In-Clinic Training

Participants were trained to use the CKD-PD app with NFC and OCR and home monitoring equipment in the PD clinic by research staff and PD nurses, starting with the CKD-PD app downloaded onto the participant’s mobile phone and account registration. Research assistants demonstrated the use of the CKD-PD app, followed by instructions on how to use the NFC and home monitoring equipment, how the uploaded hydration metrics can be monitored by the PD clinic, and how they can self-monitor at home on the app. A set of NFC hydration metric data collection equipment (body weight scale, NFC card, NFC card reader and connectors, and blood pressure machine) was prepared for each participant to use at home. They were trained to set up the equipment using a teaching video (Multimedia Appendix 2) followed by hands-on practice with a research assistant. During the study period, the participants were instructed to use the NFC data collection system and equipment, in addition to the standard method of recording their hydration metrics in a logbook.

#### Unified Theory of Acceptance and Use of Technology Survey

A structured interview questionnaire based on the Unified Theory of Acceptance and Use of Technology (UTAUT) model was completed at the beginning and end of each phase to collect perceptions of user technology acceptance and usability [26]. The questionnaire has 6 domains, each with 3 questions representing different factors affecting technology use and adoption. It was translated into the Thai language, and each question was scored on a 5-point Likert scale, with 1=strongly disagree and 5=strongly agree. The final score for each domain is the sum of the scores of the 3 questions for that domain and ranges from 3=strongly disagree to 15=strongly agree. The questionnaire was explained by the research staff and self-administered by the participants (Multimedia Appendix 3).

#### Home Observation

Research staff conducted the home observation of participants using the CKD-PD app with NFC and OCR using an observation guide (Multimedia Appendix 4), using the *think-aloud* method [27]. They asked the participants what they *liked and disliked* about each task and feature. Participants were asked to rate each feature as follows: 1=good, 2=neutral, and 3=not good. This information was recorded by research assistants using handwritten notes and summarized for use by the app improvement team.

#### Completion of Hydration Metrics

During the 2-week home use period, the number of times the participant successfully uploaded each of the required hydration metric values (body weight, blood pressure, and use of the CKD-PD app) was recorded. Participants using CAPD required 4 dialysate exchange volumes per day, whereas those using APD only required 1 each day; thus, the number of required hydration metrics varied among participants. Successful completion of hydration metrics included the entry of hydration metrics into the app and accurate upload to the CKDNET database.

#### Contact With PD Clinic

The number of times the participants contacted the PD clinic during all 3 phases was collected, along with the reason for contact.

#### Validation of Hydration Metrics

The hydration metrics collected by each participant were validated by comparing the values uploaded to the CKDNET database with the results recorded in their logbooks during the study period. Participants sent screenshots from the app, or the research staff reviewed the data on the participant’s smartphone to confirm the correct data entry in the CKD-PD app. Successful validation of hydration metrics was defined by the completion of data entry by the participant and agreement between values recorded in their logbook and the uploaded results.

#### Observation of Logbook Use

Participants were observed while entering hydration metrics in their logbooks. They were asked what they *liked and disliked* about each task and feature and rated each feature as 1=good, 2=neutral, and 3=not good.

### Ethics Approval

This study was approved by the Ethics Committee for Human Research, Faculty of Medicine, Khon Kaen University, Thailand (project number HE621494), and the Mass General Brigham institutional review board (protocol number 2019P002648). All participants provided written informed consent in the Thai language.

## Results

### Participant Characteristics

Phase 1 participants characteristics had a mean age of 46 years (SD 10.3) and a mean time on PD of 5.5 years (SD 3.8); were female (2/5 40%); used the PD method of CAPD (3/5, 60%) or APD (2/5, 40%); and had educational backgrounds of high school (2/5, 40%), bachelor’s degree (1/5, 20%), and postbachelor’s degree (2/5, 40%).

Phase 2 participant characteristics had a mean age of 48 years (SD 15.7) and a mean time on PD of 1.3 years (SD 2.5); were female (3/5, 60%); used the PD method of CAPD (1/5, 20%) or APD (4/5, 80%); and had educational backgrounds of high school (2/5, 40%), bachelor’s degree (2/5, 40%), and postbachelor’s degree (1/5, 20%). One of the group 2 participants did not complete phase 3 because of sudden death.

In comparison, the characteristics of patients on PD at Srinagarind Hospital were as follows: mean age 49 years (SD 13); mean time on PD 3 years (SD 29.7); female (33/74, 45%); PD method CAPD (42/74, 57%) or APD (32/74, 43%); and educational background of primary school (37/74, 50%), high school (12/74, 16%), bachelor’s degree (23/74, 31%), and postbachelor’s degree (2/74, 3%).

### UTAUT Survey

The UTAUT survey scores for each participant are presented in Table 2 as the total score (sum of all 6 domains) and the scores for each domain. The difference in the total score between the beginning of phase 1 or phase 2 and the end of phase 3 is also presented. Detailed results, including the scores at the beginning and end of all phases, are available in Multimedia Appendix 5.

**Table 2:** Difference in Unified Theory of Acceptance and Use of Technology total scores^a^ by participant and domain between the beginning of phase 1 or 2 and end of phase 3 (detailed results in Multimedia Appendix 5).

Participant number^b^			1		2		3		4		5		6		7		8		9		10		Values, mean
**Total score**																							
	Phase 1 or 2	89		63		86		81		87		79		66		75		71		77		77	
	Phase 3	74		76		80		74		77		72		N/A^c^		80		79		72		76	
	Difference	−15		13		−6		−7		−10		−7		N/A		5		8		−5		−2.7	
**Difference by domains**																							
	Performance expectancy	−2		3		1		1		−1		−1		N/A		1		1		0		0.33	
	Effort expectancy	−2		4		1		−1		1		−1		N/A		0		0		1		0.33	
	Social influence	−3		3		1		0		−2		−1		N/A		−1		1		−1		−0.67	
	Voluntariness	−3		−3		−5		−2		−3		−3		N/A		2		3		−4		−2	
	Intention to use	−2		3		−4		−4		−6		0		N/A		2		3		0		−0.9	
	Facilitating conditions	−3		3		0		−1		1		−1		N/A		1		0		−1		−0.1	

^a^Scores for individual questions from 1 (strongly disagree) to 5 (strongly agree). The domain scores ranged from 5 to 15. The total score for all the domains ranged from 30 to 90.

^b^Participants 1 to 5: phase 1 and phase 3; participants 6 to 10: phase 2 and phase 3.

^c^N/A: not applicable; participant 7 expired before phase 3.

Of the 9 participants who completed phase 1 or 2 and phase 3, 6 (67%) individuals had a decrease in the total UTAUT score, ranging from −5 to −15 points. There were 33% (3/9) of individuals who had an increase in the total UTAUT score, ranging from 5 to 13 points. The mean score at the beginning of phases 1 or 2 was 77 (SD 8.8), with a range of 63 to 89. The mean score at the end of phase 3 was 76 (SD 3.2), with a range of 72 to 80. In group 1, the domains of voluntariness (−3) and intention to use (−2.6) showed the largest decrease in mean difference between the beginning of phase 1 and the end of phase 3, with all participants reporting a negative score in voluntariness and 80% (4/5) of participants reporting a negative score in intention to use. In group 2, there were small decreases in voluntariness (−0.5), social influence (−0.5), and facilitating conditions (−0.25) and an increase in intention to use (1.25) between phases 2 and 3 (Multimedia Appendix 5).

### Participant Observation

The home observation results for each participant using the CKD-PD app with the NFC and OCR and monitoring system during the home observations are summarized by phase for all participants in Table 3, with detailed responses in Multimedia Appendix 6. Feature ratings are provided for each phase. The participants rated almost all features as *good*, except for some data entry tasks using NFC, which were rated as *neutral*. Participants liked automatic data entry with NFC; however, technical issues such as slow transfer of time and difficulty tapping the card resulted in lower scores. Participants were asked to rate the entry of body weight and dialysate volume using NFC at the start of phase 3 based on their experience in phases 1 or 2 for comparison with manual data entry used in phase 3. The average rating for data entry with NFC was 2.3 for each task compared with 1.1 for manual data entry (Multimedia Appendix 6). Scores for all tasks using the handwritten logbook were nearly all higher than the CKD app with or without NFC and OCR, consistent with the preference for using the app.

**Table 3:** Feature and task ratings^a^ from participant observation in phases 1, 2, and 3 (detailed results in Multimedia Appendix 6).

Feature	Phase 1^b^, mean (range)	Phase 2^c^, mean (range)	Phase 3^d^, mean (range)	Phase 3^e^, mean (range)
Open screens	1.1 (1-2)	1 (1)	1.6 (1-3)	N/A^f^
Enter body weight	1.1 (1-2)	1.2 (1-2)	1.1 (1-2)	1.4 (1-2)
Enter blood pressure	1.1 (1-2)	1.6 (1-3)	1.1 (1-2)	1.4 (1-3)
Enter dialysate	1.1 (1-2)	1.2 (1-2)	1.1 (1-2)	1.8 (1-3)
View metrics	1.3 (1-2)	1 (1)	1.1 (1-2)	1.7 (1-3)
Interpret metrics	1.1 (1-2)	1.2 (1-2)	1.1 (1-2)	N/A
Communications	1 (1)	1 (1)	1 (1)	1.2 (1-3)
User incentives	1.1 (1-2)	1.2 (1-2)	1.2 (1-3)	1.9 (1-3)

^a^Rating of task or feature: 1=good, 2=neutral, and 3=not good.

^b^Group 1 using the chronic kidney disease–peritoneal dialysis app with near-field communication or optical character recognition data entry; participants 1 to 5.

^c^Group 2 using the chronic kidney disease–peritoneal dialysis app with near-field communication or optical character recognition data entry; participants 6 to 10.

^d^Group 1 and 2 using the chronic kidney disease–peritoneal dialysis app with manual entry; participants 1 to 10, excluding 7.

^e^Group 1 and 2 using logbook; participants 1 to 10, excluding 7.

^f^N/A: not applicable.

In general, participants found that manual data entry was easier to perform than using the NFC and OCR system. The use of NFC and OCR presented multiple challenges. Some of these issues were solved by providing tools such as an extension device for tapping the card to eliminate the need to bend over and touch the card to the scale on the floor and training participants to wait long enough for data transfer. Some issues were more difficult to troubleshoot within the time frame of this study, such as (1) lack of space in participants’ homes for NFC setup and access to power outlets; (2) internet instability resulting in slow data transfer; (3) size and design of the NFC-assembled weight scale, making it difficult for older or obese patients to stand on; and (4) lack of an alert to indicate that the NFC device was ready to place the NFC card for reading. Most patients also did not like to use the OCR function to enter the blood pressure readings because of poor image clarity and variations in ambient home lighting conditions.

Several important issues related to the functionality of the CKD-PD app were detected and corrected. The dialysate in and out volumes were reported by PD cycle, and the net daily ultrafiltration volume was not accurately calculated and displayed. There were issues with the display of hydration metrics, such as incorrect scale on the graph and a lack of previous results for comparison. During home observation of participants using the CKD-PD app with the NFC and OCR and monitoring system, participants expressed concerns about the usability of the app. An example was slowness when opening each icon, which was determined to be from opening multiple apps at once, and slow cellular or Wi-Fi network speeds were contributing factors. BMI was misinterpreted as *overweight* because of translation issues between the English and Thai languages. This was corrected by changing the wording so it would not be confused with *weight*. Participant height had to be entered daily to calculate the BMI. As this measurement did not change, the app was modified so that it automatically entered the participant height obtained daily. The original font was small and difficult to read. This was adjusted, and the readability of the screens was improved.

### Completion and Validation of Hydration Metrics

The percentage completion of the hydration metrics for each participant was collected for all 3 phases (Table 4). The mean percentages of completion in phases 1 and 2 were 88% (SD 19) and 83% (SD 6.3), respectively, with a range of 57% to 100%. This was compared with the mean percentage of completion in phase 3 for all participants (68%, SD 18.6, range 18%-100%; Multimedia Appendix 7). The most common reasons for not collecting the hydration metrics in phases 1 and 2 were not knowing how to enter the data, forgetting to enter the data, problems with the system uploading the data to the CKDNET database, and switching from Android to iOS systems. The reasons for incomplete hydration metric collection in phase 3 (not using NFC) were forgetting to send the data to the CKDNET and switching from Android to iOS.

**Table 4:** Percentage completion of hydration metrics (detailed in Multimedia Appendix 7).

Participant	Phase 1^a^		Phase 2^b^		Phase 3^c^		Difference (%)^d^
	Total, N	Done, n (%)	Total, N	Done, n (%)	Total, N	Done, n (%)	
1	84	80 (95)	—^e^	—	42	19 (45)	−50
2	66	39 (59)	—	—	84	42 (50)	−9
3	42	42 (100)	—	—	42	26 (62)	−38
4	84	73 (87)	—	—	84	79 (94)	7
5	48	48 (100)	—	—	56	40 (71)	−29
6	—	—	42	38 (91)	42	42 (100)	9
7^f^	—	—	39	35 (90)	N/A^g^	N/A	N/A
8	—	—	42	37 (88)	42	39 (93)	5
9	—	—	30	24 (57)	84	15 (18)	−39
10	—	—	80	27 (90)	42	33 (79)	−11
Values, mean	65	56 (88)	46.6	32 (83)	57.6	37 (68)	−15

^a^Phase 1: participants 1 to 5.

^b^Phase 2: participants 6 to 10.

^c^Phase 3: participants 1 to 10.

^d^Difference in percentage completion between phase 1 or 2 and phase 3.

^e^Not available.

^f^Participant 7 expired before completion of the study.

^g^N/A: not applicable.

Of the 9 participants who entered hydration metric data in phase 1 or 2 and phase 3, there were 4 participants who had a greater than 29 percentage point decrease in their hydration metric completion. The other 5 participants had minimally decreased or increased hydration metric completion, ranging from −11% to 9%.

Validation of the hydration metrics improved from phases 1 or 2 and phase 3 (Table 5). In phases 1 and 2, the main issues were the upload of inaccurate body weight and ultrafiltration metric results to the CKDNET. This was because of problems with the NFC weight scale and app calculation of the ultrafiltration volume. In phase 3, these issues were resolved, the hydration metrics were fully validated with participants correctly entering the data in the CKD-PD app, and the results entered were accurately uploaded to the CKDNET.

**Table 5:** Validation of hydration metrics.

Participant			1		2		3		4		5		6		7		8		9		10		Values, n (%)^a^
**Phase 1^b^**																							
	Body weight	No^c^		No		No		No		No		—^d^		—		—		—		—		0 (0)	
	Blood pressure	Yes^e^		Yes		No		Yes		Yes		—		—		—		—		—		4 (80)	
	Dialysate in	Yes		No		Yes		No		Yes		—		—		—		—		—		3 (60)	
	Dialysate out	Yes		No		Yes		No		Yes		—		—		—		—		—		3 (60)	
	Ultrafiltration volume	Yes		No		Yes		No		No		—		—		—		—		—		2 (40)	
**Phase 2^f^**																							
	Body weight	—		—		—		—		—		Yes		No		Yes		No		Yes		3 (60)	
	Blood pressure	—		—		—		—		—		Yes		Yes		Yes		Yes		Yes		5 (100)	
	Dialysate in	—		—		—		—		—		Yes		Yes		Yes		Yes		Yes		5 (100)	
	Dialysate out	—		—		—		—		—		Yes		Yes		Yes		Yes		No		4 (80)	
	Ultrafiltration volume	—		—		—		—		—		Yes		No		Yes		Yes		No		3 (60)	
**Phase 3^g^**																							
	Body weight	Yes		ND^h^		ND		Yes		Yes		Yes		N/A^i^		Yes		Yes		Yes		7 (100)	
	Blood pressure	Yes		Yes		Yes		Yes		Yes		Yes		N/A		Yes		Yes		Yes		9 (100)	
	Dialysate in	Yes		Yes		Yes		Yes		Yes		Yes		N/A		Yes		Yes		Yes		9 (100)	
	Dialysate out	Yes		Yes		Yes		Yes		Yes		Yes		N/A		Yes		Yes		Yes		9 (100)	
	Ultrafiltration volume	Yes		Yes		Yes		Yes		Yes		Yes		N/A		Yes		Yes		ND		9 (100)	

^a^Number and percentage of hydration metric values entered and uploaded with the correct results to Chronic Kidney Disease Prevention in the Northeast of Thailand.

^b^Phase 1: participants 1 to 5.

^c^Not uploaded accurately to Chronic Kidney Disease Prevention in the Northeast of Thailand.

^d^Not available.

^e^Uploaded accurately to Chronic Kidney Disease Prevention in the Northeast of Thailand.

^f^Phase 2: participants 6 to 10.

^g^Phase 3: participants 1 to 10.

^h^ND: not done by participant.

^i^N/A: not applicable; participant 7 expired before completion of the study.

### Clinic Contacts

The number of times each participant contacted the PD clinic during each phase is presented in Table 6. All contacts were made using the Line messaging app by chat, audio, or video calls, except for1 in-person contact during phase 1. In phase 1, there were 104 clinic contacts compared with 13 and 22 in phases 2 and 3, respectively, and most were related to issues using the CKD-PD app. In phase 3, 59% (13/22) of the contacts were related to clinical concerns.

**Table 6:** Number and reason for contacting the peritoneal dialysis clinic by phase.

Phase	Total number of contacts, N	Clinical issue, n (%)	App issue, n (%)	Other, n (%)
Phase 1	104	0 (0)	99 (95.2)	5 (4.8)
Phase 2	13	3 (23.1)	10 (76.9)	0 (0)
Phase 3	22	13 (59.1)	9 (40.9)	0 (0)

### Improvement Team Activities

After the completion of each phase, the research team summarized the findings for the app improvement team (Multimedia Appendix 8). In phase 1, troubleshooting issues with NFC and OCR were the focus of the improvement activities, along with app design issues. In phase 2, additional work was performed with the NFC data entry; however, more focus was given to resolving issues with how the hydration metric data were displayed and uploaded to the CKDNET database. In phase 3, the NFC prototype was not used as the participants preferred to input data manually into the CKD-PD app, and the upload of the hydration metric data to the CKDNET database was not reliable using NFC. The app improvement team made final improvements to improve the stability of the data transfer from the CKD-PD app to the CKDNET database, added the total daily ultrafiltration volume to the CKDNET, and set parameters for overhydration alerts.

## Discussion

### Principal Findings

#### Overview

mHealth apps are touted as having great potential to transform the delivery of health care; however, the real-world development of mHealth apps in low- and middle-income countries reveals few success stories [12,17]. Often, a good app idea fails to succeed as the design does not meet the users’ needs and is difficult to use. Our research describes how a rapid cycle process improvement strategy helped researchers understand the benefits and challenges of using an mHealth app for use by patients on PD in a low-resource setting and optimize its features. The process revealed valuable insights into the factors influencing user attitudes, identifying technological and design flaws, and addressing barriers to user adoption of the CKD-PD app and monitoring system. Moreover, we believe that this methodology can be accomplished without requiring significant training in data collection, statistical analysis, or funding. The general principles of the user design process are applicable to a wide variety of locations, contexts, and subject domains.

#### User Adoption

The total scores for all the participants for most domains were in the *agree* and *strongly agree* categories, demonstrating a strong willingness to adopt the technology at the beginning of the study. Over the course of the study, 67% (6/9) of participants who completed both assigned phases demonstrated decreased interest in using the technology, although the total scores remained in the *agree* range or above. The decreases in the total score were primarily driven by decreases in the domains of voluntariness and intention to use. We believe this is likely because of issues with the NFC and OCR data entry features and app speed and functionality. We acknowledge that the UTAUT questionnaire is not typically used to evaluate technology adoption over time; however, we found that it provided insights into how our participants’ views changed over the course of using the CKD-PD app and monitoring system, although they had limited generalizability. The small sample size limited the statistical validity of our findings. However, we found the information useful as a *sentiment analysis* regarding user experience and provided insights into how different individuals may adopt the CKD-PD app.

#### CKD-PD App Issues

Design issues centered on simplicity and ease of use compared with a pencil and paper logbook. Simplifying the use of the app, for example, opening the app and the organization of the screens, readability of the fonts, input of the data, and graphical representation of trends, clearly needed to be refined based on user feedback. PD requires a substantial commitment of time to collect and record data from patients and their families throughout the day, whether in a logbook or an app. Our participants generally reacted positively to the self-monitoring features and quickly engaged with this new functionality but became frustrated when some features did not work well for them.

#### Technology Issues

Technology issues included problems with the NFC devices, unstable internet access, slow internet speed, app stability, uploading data to the CKD-NET cloud, and software issues causing it to freeze or not upload data properly. The participant observation revealed that NFC and OCR were good solutions for automatic data entry but that they need to be seamlessly integrated into the measurement devices; for example, the location of NFC on the scales on the floor was inconvenient, slow data transfer, and poor quality image capture with OCR. Despite these barriers, participants liked the concept, and with further development, we believe that NFC and OCR could be simple and effective methods of mitigating the barrier of data entry [28,29], as has been done with other devices such as glucose monitors.

Internet access, especially in low-income rural areas, is frequently slow or unstable, making the use of digital apps cumbersome. Some patients were concerned about the cost of sending or receiving data, although internet access was subsidized for the participants in this study. These will be challenging issues to remedy; however, if successful in reducing complications from overhydration, it may prove cost-effective in the long run to provide financial support for internet access for patients on PD, given the alternative costs of not using the app.

Programming issues resulting in app instability and data upload errors were critical problems that were identified. These were fixable by our software engineers and resulted in improved validation of the hydration metrics in phase 3. The completion of the hydration metrics dropped between phases 1 and 3. We believe this occurred as participants in phases 1 and 2 used NFC, which automatically uploaded the hydration metrics, and decreased after the team elected not to use NFC and OCR in phase 3 because of usability issues and inaccurate upload of the hydrate metrics to the CKDNET with NFC and OCR. The hydration metric accuracy improved with this change and resulted in 100% validation of the hydration metrics in phase 3. However, when manual entry was used in phase 3, participants had to remember to upload their data, resulting in incomplete hydration metrics. In the final improvements, a reminder alert addressed this issue.

#### Communication Issues

Contact with the PD clinic was much more frequent in phase 1 than in phase 2, although the participants in both phases were new users of the app. The study staff felt that this was because of differences in individual comfort when using the CKD-PD app. In phase 3, there were fewer contacts about the app, although there were 10 participants compared with 5 in phases 1 and 2. The contacts in phase 3 were about clinical concerns, suggesting increased confidence in self-monitoring features. In general, participants gave high ratings and positive comments about the ease of communication with the PD clinic using the CKD-PD app.

#### Impact on Future Deployment and Development of CKD-PD

Our research findings provided critically useful information for the optimization of the CKD-PD app and monitoring system. We plan to use it in a randomized controlled trial to further evaluate the efficacy of CKD-PD in the early detection and treatment of overhydration.

We have developed training materials for CKD-PD app users based on the insights gained from this study and plan to share them with future CKD-PD app users. We have improved awareness of user adoption issues and realize that this will be an iterative process as more patients on PD use the app. We believe that with additional engineering and design work, NFC is a potential solution for automating data entry, and we plan to pursue the further development of these features.

### Limitations

Our study has several limitations. The NFC system and CKD-PD app were initially tested together; however, the NFC issues were too complicated to solve within the time frame and budget of the study period. Through targeted observations and data collection, we were able to tease out NFC and data entry issues from the app design and cloud-based monitoring processes.

We identified potential sources of bias. Observation bias could have influenced our results as participant observations were conducted by research staff from the PD clinic. Patients on PD, cared for by nephrologists and PD nurses from their clinic, may be inclined to report feedback they thought the PD staff wanted to hear. Confirmation bias may also be a concern as research staff may be looking for patterns or use issues that confirm opinions they already hold about the CKD-PD app, NFC and OCR data entry, and the monitoring system. Our sample size was small; however, this is consistent with recommendations for user design studies, indicating that a few participants can uncover most usability issues [30]. We also acknowledge the potential for selection bias in our participants, given their higher educational level than the average population in rural northeast Thailand, although the participants were representative of the Srinagarind Hospital PD patient population. We selected participants who had baseline comfort with smartphones for the user design study, although it limits generalizability as they were more interested and engaged in the design process.

An additional weakness is that we did not conduct an in-depth investigation into why our participants responded to the UTAUT questionnaire as much as they did. Although it has been validated and used in Thai populations [26,31], we did not consider the impact of age, gender, education, and cultural influences on UTAUT scores [32,33]. We acknowledge that UTAUT results have limited generalizability.

### Comparison With Prior Work

Our research included several recommended strategies for mHealth app development and evaluation [34,35]. Our iterative rapid cycle process improvement approach used a multidisciplinary team, including patients, computer engineers and programmers, nephrologists, and PD nurses, to evaluate the CKD-PD app. We conducted our study activities in the participants’ homes and PD clinics where it would be used [36,37]. Our structured observation using the think-aloud method provided the app improvement team with a broad sense of what worked and what did not work, in addition to specific actionable feedback. Finally, our study participants were selected from the same study population as those who will use the CKD-PD app in northeast Thailand.

Our research team found several critical usability and functionality issues during the study. This experience reinforces the importance of these steps before an mHealth app can be successfully implemented [12,13,17]. Although our current NFC and OCR data entry features are not optimized, our results support the idea that app users want these features [27].

### Conclusions

In conclusion, using rapid cycle progress improvement methodologies with a multidisciplinary team proved to be a useful strategy for optimizing the CKD-PD app and monitoring system. Our experience aligns with insights and recommendations regarding mHealth technology design and evaluation that using a multidisciplinary team in the context of the system in which it is used is essential for successful deployment. This process revealed critical issues that once addressed, position the CKD-PD app and monitoring system to achieve its potential to improve health outcomes.

## Appendix

Multimedia Appendix 1 Chronic Kidney Disease–Peritoneal Dialysis app screenshots.

Multimedia Appendix 2 Using Chronic Kidney Disease–Peritoneal Dialysis app (near-field communication version).

Multimedia Appendix 3 Unified Theory of Acceptance and Use of Technology questionnaire on adoption and use of the Chronic Kidney Disease–Peritoneal Dialysis app.

Multimedia Appendix 4 Participant observation guide.

Multimedia Appendix 5 Unified Theory of Acceptance and Use of Technology scores by participant and domain between phases 1 and 3 or phases 2 and 3.

Multimedia Appendix 6 Summary of participant observation phases 1, 2, and 3.

Multimedia Appendix 7 Detailed hydration metric completion.

Multimedia Appendix 8 Recommendations from the app improvement team.

## Abbreviations

APD: automatic peritoneal dialysis
CAPD: continuous ambulatory peritoneal dialysis
CKD: chronic kidney disease
CKDNET: Chronic Kidney Disease Prevention in the Northeast of Thailand
CKD-PD: chronic kidney disease–peritoneal dialysis
mHealth: mobile health
NFC: near-field communication
OCR: optical character recognition
PD: peritoneal dialysis
UTAUT: Unified Theory of Acceptance and Use of Technology
