# Supplementary material for: Optimization of the Chronic Kidney Disease–Peritoneal Dialysis App to Improve Care for Patients on Peritoneal Dialysis in Northeast Thailand: User-Centered Design Study
Source: JMIR Form Res. 2022 Jul 6;6(7):e37291. doi: 10.2196/37291 (PMC9301552; doi:10.2196/37291)

Multimedia Appendix 1: CKD-PD app Screenshots

1. CKD-PD mobile application "CKD รักษ์ไต"
is available for free download in both Android and iOS format. There is also a CKD app for individuals that are not on peritoneal dialysis but want to track their own renal status, blood pressure, and diet. It is not monitored through the PD clinic

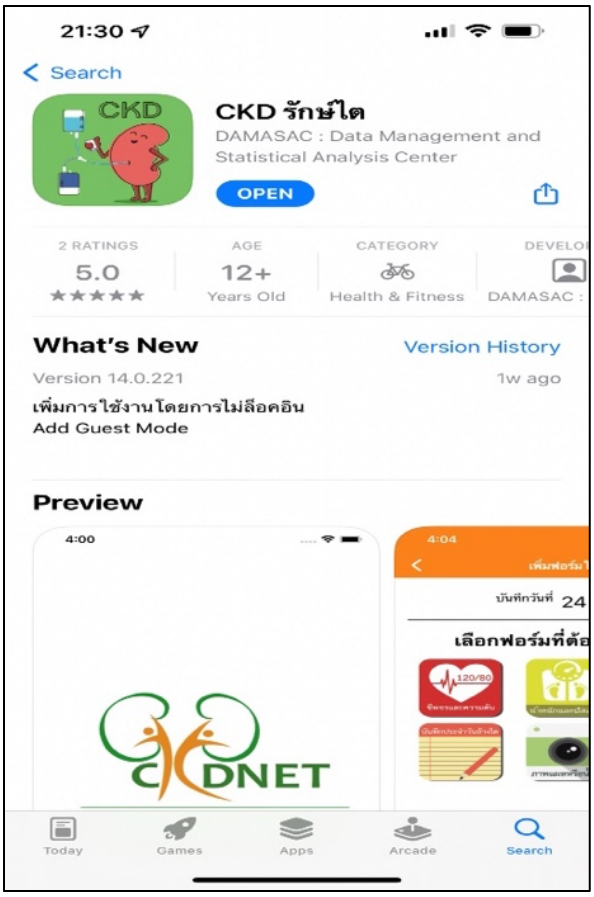

2. Patients are enrolled in the CKD-PD monitoring system through the PD clinic. PD nurses provide a user name and password. Once enrolled, their hydration metrics can be uploaded to the CKDNET data base on the Thai Care Cloud. This allows the PD clinic staff to monitor their hydration metrics from the PD clinic.

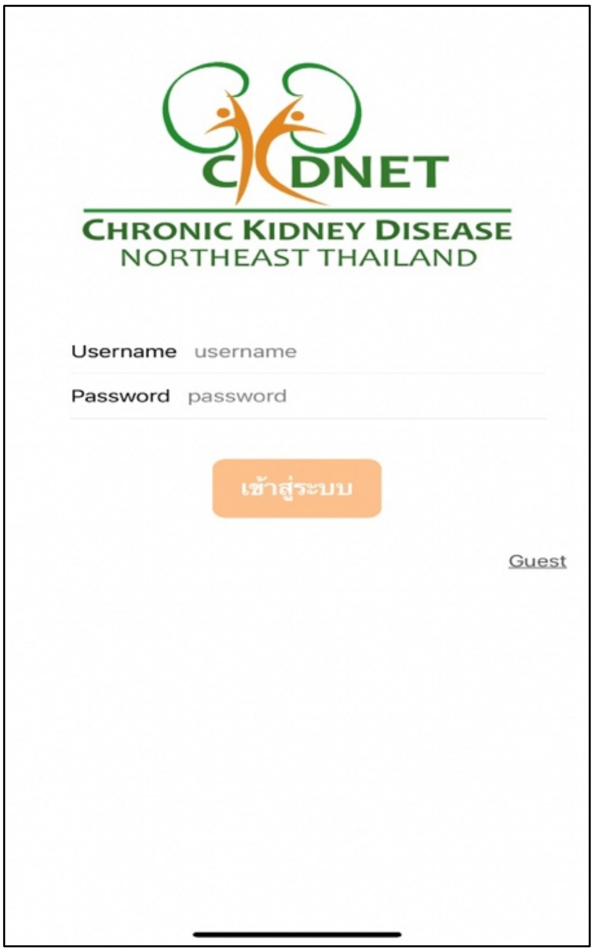

3. The home screen has patient demographics and hydration metric data. New hydration metric data can be added by using the “+” key.

The information displated includes

- Patients identification
- Patient’s daily hydration matric data
    - Peritoneal dialysis fluid (PDF) instilled and drained for each cycle
    - Body weight
    - Blood pressure and heart rate
    - Fluid intake
    - Urine volume

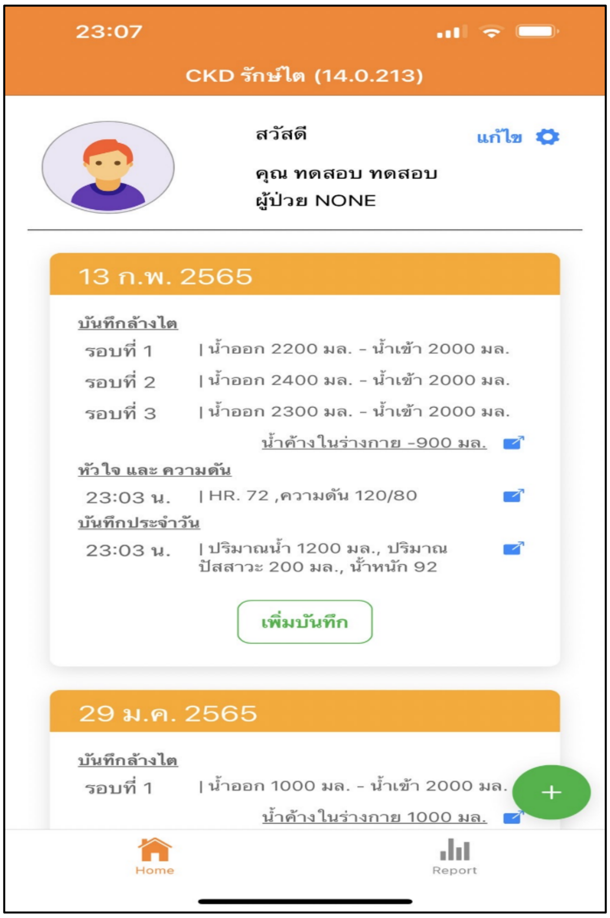

4. Patients can view and record each hydration metric separately.

- Blood pressure and heart rate
- Body weight, fluid intake and urine
- Blood sugar level
- PDF instilled and drained volume

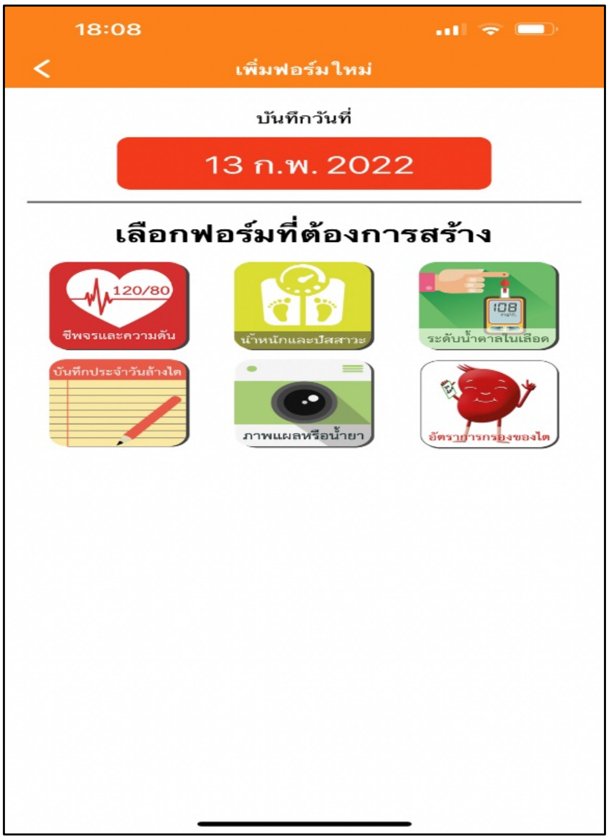

4.1 Blood pressure and heart rate are entered on the same screen. Data entry can be done by typing or voice recognition.

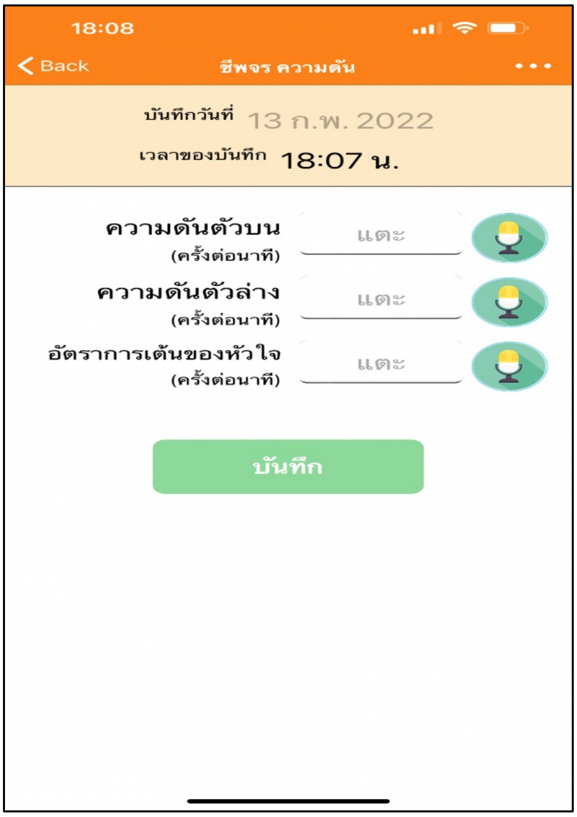

4.2 Body weight, height, fluid intake and urine volume are recorded on a separate screen. Manual and voice data entry are available.

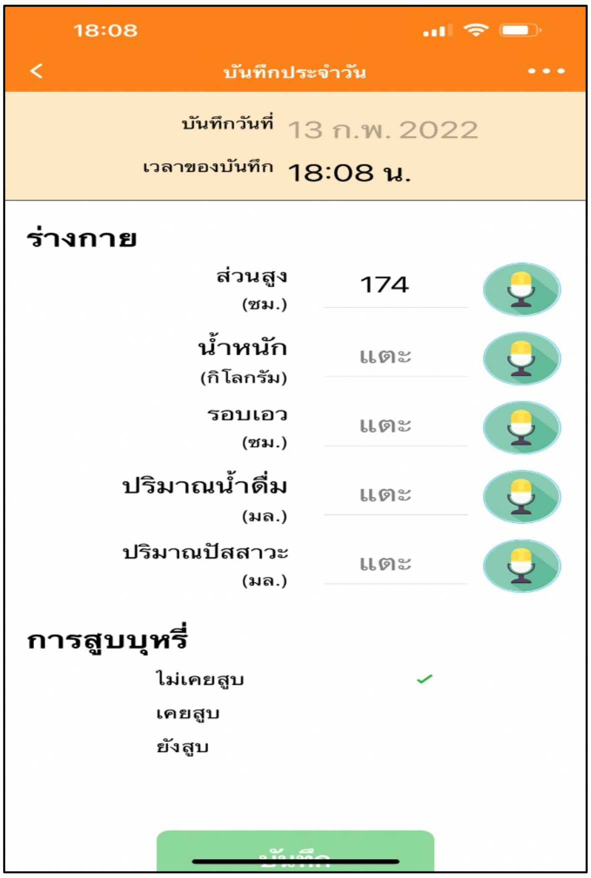

4.3 The peritonal fluid volumes instilled and drained are recorded on the next screen. In addition to fluid volume, time of instillation and drainage and cycle time are recorded. There are manual and voice data entry options.

PD patients do multiple cycles each day"+" icon for the next cycle. At the end of the 24 hour period, the total fluid voulme removed or ultrafiltration volume is automatically calculated by using the arrow icon.

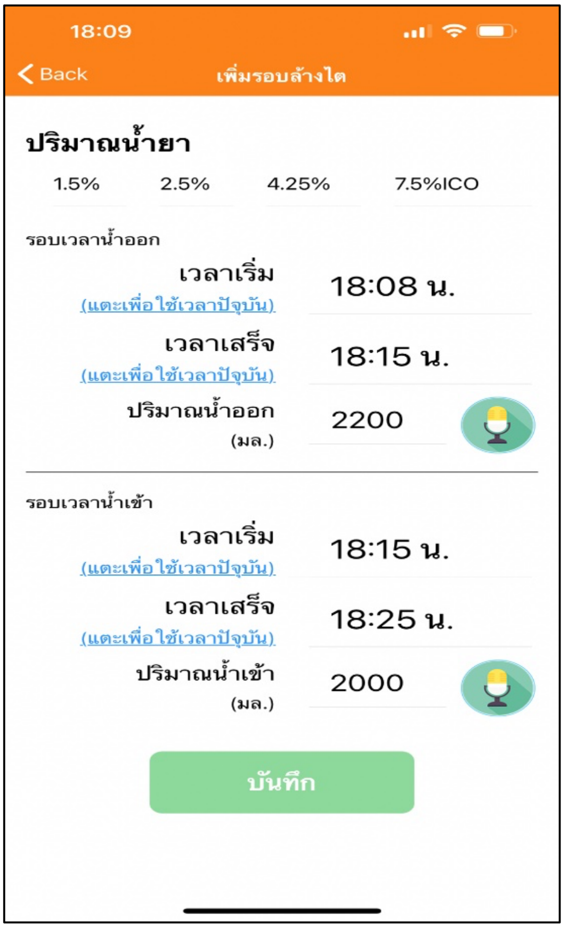

5. Patients can monitor their hydration metrics over a 2 week period using the report function screens. There are control charts showing the acceptable ranges that have been personalized by the nephrologists for each patient. Patient can monitor their hydration metrics at home and contact the PD clinic if they are out of range.

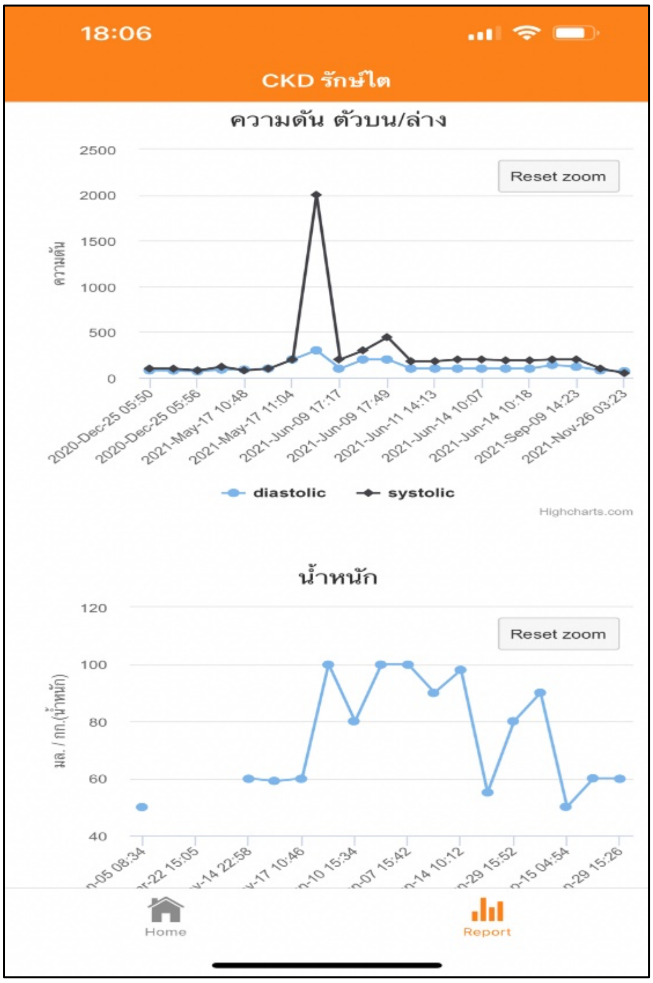

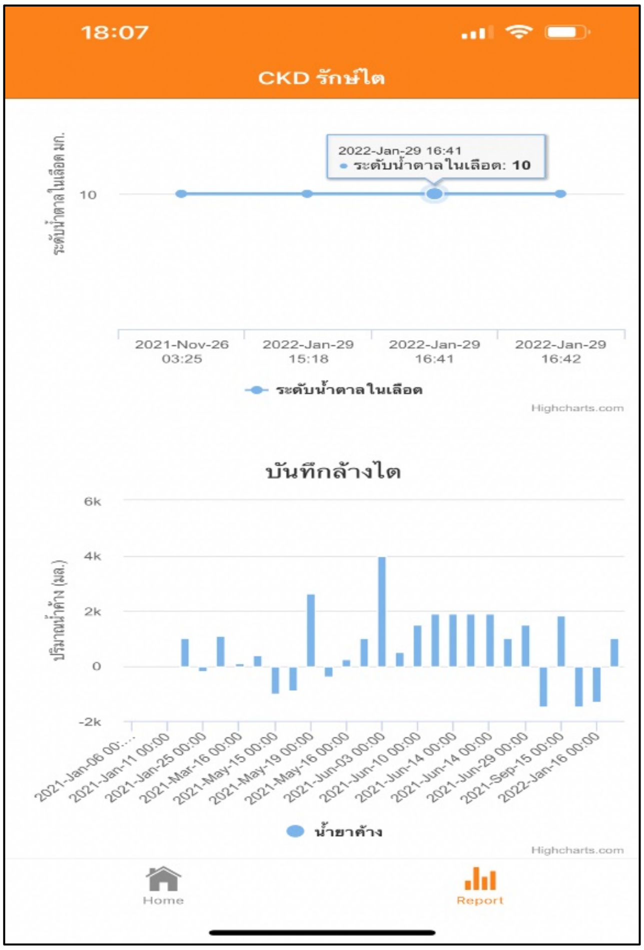

Supplement: Multimedia Appendix 1 [file formative_v6i7e37291_app1.pdf]
