# Supplementary material for: Optimization of the Chronic Kidney Disease–Peritoneal Dialysis App to Improve Care for Patients on Peritoneal Dialysis in Northeast Thailand: User-Centered Design Study
Source: JMIR Form Res. 2022 Jul 6;6(7):e37291. doi: 10.2196/37291 (PMC9301552; doi:10.2196/37291)
Supplement: Multimedia Appendix 3 [file formative_v6i7e37291_app3.pdf]

Multimedia Appendix 4: UTAUT Questionnaire on Adoption and Use of the CKD-PD app

These are statements about the CKD-PD app and how to use it. We are interested in your thoughts and expectations about using this new technology. Please select the answer that best represents your agreement with the statement. There are no right or wrong answers.

Strongly agree = 5; Agree = 4; Neutral = 3; Disagree = 2; Strongly disagree =1

Group 1: Performance expectancy

1. The CKD-PD app will be useful for managing my peritoneal dialysis (PD) and health.

____Strongly agree ____Agree ____Neutral ____Disagree _____Strongly disagree

2. Using the CKD-PD app will be easier than recording information in my PD logbook.

____Strongly agree ____Agree ____Neutral ____Disagree _____Strongly disagree

3. If I use the CKD-PD app, my health will improve.

____Strongly agree ____Agree ____Neutral ____Disagree _____Strongly disagree

Group 2: Effort expectancy

1. Learning to use CKD-PD will be easy for me.

____Strongly agree ____Agree ____Neutral ____Disagree _____Strongly disagree

2. It will be easy for me to become skillful at using the CKD-PD app.

____Strongly agree ____Agree ____Neutral ____Disagree _____Strongly disagree

3. It is easy to understand how the CKD-PD app works and how the features can help me.

____Strongly agree ____Agree ____Neutral ____Disagree _____Strongly disagree

Group 3: Social influence

1. My family thinks it is important for me to use CKD-PD.

____Strongly agree ____Agree ____Neutral ____Disagree _____Strongly disagree

2. The PD clinic staff supports the use of the CKD-PD app.

____Strongly agree ____Agree ____Neutral ____Disagree _____Strongly disagree

3. People who influence my behavior think it is important for me to use the CKD-PD app.

____Strongly agree ____Agree ____Neutral ____Disagree _____Strongly disagree

Group 4: Voluntariness

1. My doctor does not require me to use the CKD-PD app.

____Strongly agree ____Agree ____Neutral ____Disagree _____Strongly disagree

2. The PD clinic staff does not require me to use the CKD-PD app.

____Strongly agree ____Agree ____Neutral ____Disagree _____Strongly disagree

3. My family does not require me to use the CKD-PD app.

____Strongly agree ____Agree ____Neutral ____Disagree _____Strongly disagree

Group 5: Intention to use

1. I intend to use the CKD-PD app all the time.

____Strongly agree ____Agree ____Neutral ____Disagree _____Strongly disagree

2. I predict I will use the CKD-PD app all the time.

____Strongly agree ____Agree ____Neutral ____Disagree _____Strongly disagree

3. I plan to use CKD-PD all the time.

____Strongly agree ____Agree ____Neutral ____Disagree _____Strongly disagree

Group 6: Facilitating conditions

1. I have knowledge and resources to learn to use the CKD-PD app.

____Strongly agree ____Agree ____Neutral ____Disagree _____Strongly disagree

2. I will be able to get help to use CKD-PD if needed.

____Strongly agree ____Agree ____Neutral ____Disagree _____Strongly disagree

3. The CKD-PD app fits well with the way I do my peritoneal dialysis.

____Strongly agree ____Agree ____Neutral ____Disagree _____Strongly disagree
