# Supplementary material for: Optimization of the Chronic Kidney Disease–Peritoneal Dialysis App to Improve Care for Patients on Peritoneal Dialysis in Northeast Thailand: User-Centered Design Study
Source: JMIR Form Res. 2022 Jul 6;6(7):e37291. doi: 10.2196/37291 (PMC9301552; doi:10.2196/37291)
Supplement: Multimedia Appendix 4 [file formative_v6i7e37291_app4.pdf]

Multimedia Appendix 4: Participant Observation Guide				
Task/feature	Prompt 1<br>What do you **like** about this feature?	Prompt 2<br>What do you **dislike** about this feature?	[a]Rating	[b]Observations
Opening and accessing screens				
Entering daily body weight				
Entering blood pressure				
Entering dialysate volume				
Viewing hydration metrics				
Interpreting hydration metrics				
Communication with PD clinic				
User incentives				

[a] Rating: 1=Good, 2=Neutral, 3=Not good

[b]Observations by research assistant while participant is performing task
