# Supplementary material for: Optimization of the Chronic Kidney Disease–Peritoneal Dialysis App to Improve Care for Patients on Peritoneal Dialysis in Northeast Thailand: User-Centered Design Study
Source: JMIR Form Res. 2022 Jul 6;6(7):e37291. doi: 10.2196/37291 (PMC9301552; doi:10.2196/37291)
Supplement: Multimedia Appendix 5 [file formative_v6i7e37291_app5.pdf]

Multimedia Appendix 5: UTAUT Scores[a] by Participant and Domain Between Phase 1 and 3, or Between Phase 2 and 3

Group 1	Participant 1							Participant 2							Participant 3							Participant 4							Participant 5							
Phase #	1			3				1			3				1			3				1			3				1			3				
Week #	0	3	Diff[b]	16	20	Diff	**Tot diff[c]**	0	3	Diff	16	20	Diff	**Tot diff**	0	3	Diff	16	20	Diff	**Tot diff**	0	3	Diff	16	20	Diff	**Tot diff**	0	3	Diff	16	20	Diff	**Tot diff**	**Mean diff[d]**
Performance expectancy	15	15	0	14	13	-1	**-2**	9	13	4	12	12	0	**3**	14	13	-1	15	15	0	**1**	11	13	2	13	12	-1	**1**	14	14	0	14	13	-1	**-1**	**0.4**
Effort expectancy	14	15	1	12	12	0	**-2**	11	12	1	12	15	3	**4**	14	14	0	15	15	0	**1**	14	14	0	12	13	1	**-1**	14	14	0	14	15	1	**1**	**0.6**
Social Influence	15	14	-1	13	12	-1	**-3**	10	11	1	11	13	2	**3**	14	13	-1	14	15	1	**1**	13	3	-10	14	13	-1	**0**	15	15	0	15	13	-2	**-2**	**-0.4**
Voluntariness	15	9	-6	12	12	0	**-3**	9	12	3	7	6	-1	**-3**	14	9	-5	13	9	-4	**-5**	15	15	0	12	13	1	**-2**	15	15	0	15	12	-3	**-3**	**-3.0**
Intention to use	15	15	0	15	13	-2	**-2**	12	12	0	15	15	0	**3**	15	15	0	15	11	-4	**-4**	15	15	0	14	11	-3	**-4**	15	15	0	15	9	-6	**-6**	**-2.6**
Facilitating conditions	15	15	0	13	12	-1	**-3**	12	12	0	13	15	2	**3**	15	13	-2	13	15	2	**0**	13	13	0	13	12	-1	**-1**	14	15	1	14	15	1	**1**	**0**
Total score[e]	89	83	-6	79	74	-5	**-15**	63	72	9	70	76	6	**13**	86	77	-9	85	80	-5	**-6**	81	73	-8	78	74	-4	**-7**	87	88	1	87	77	-10	**-10**	**1**
**Group 2**	**Participant 6**							**Participant 7**							**Participant 8**							**Participant 9**							**Participant 10**							
Phase #	2			3				2			3				2			3				2			3				1			3				
Week #	8	12	Diff	16	20	Diff	**Tot diff**	8	12	Diff	16	20	Diff	**Tot diff**	8	12	Diff	16	20	Diff	**Tot diff**	8	12	Diff	16	20	Diff	**Tot diff**	8	12	Diff	16	20	Diff	**Tot diff**	**Mean diff**
Performance expectancy	13	13	0	13	12	-1	**-1**	12	11	-1	NA	NA	NA	**NA**	13	14	1	14	14	0	**1**	12	10	-2	13	13	0	**1**	13	13	0	14	13	-1	**0**	**0.25**
Effort expectancy	13	12	-1	13	12	-1	**-1**	12	12	0	NA	NA	NA	**NA**	12	13	1	13	12	-1	**0**	15	15	0	14	15	1	**0**	12	14	2	13	13	0	**1**	**0.5**
Social Influence	13	13	0	12	12	0	**-1**	11	13	2	NA	NA	NA	**NA**	14	14	0	15	13	-2	**-1**	12	14	2	13	13	0	**1**	13	14	1	13	12	-1	**-1**	**-0.5**
Voluntariness	14	11	-3	15	11	-4	**-3**	11	12	1	NA	NA	NA	**NA**	12	11	-1	9	14	5	**2**	9	12	3	12	12	0	**3**	13	13	0	12	9	-3	**-4**	**-0.5**
Intention to use	13	12	-1	14	13	-1	**0**	8	12	4	NA	NA	NA	**NA**	12	14	2	14	14	0	**2**	9	10	1	14	12	-2	**3**	13	14	1	14	13	-1	**0**	**1.25**
Facilitating conditions	13	12	-1	14	12	-2	**-1**	12	12	0	NA	NA	NA	**NA**	12	12	0	14	13	-1	**1**	14	13	-1	14	14	0	**0**	13	13	0	12	12	0	**-1**	**-0.25**
Total score	79	73	-6	81	72	-9	**-7**	66	72	6	NA	NA	NA	**NA**	75	78	3	79	80	1	**5**	71	74	3	80	79	-1	**8**	77	81	4	78	72	-6	**-5**	**0.25**

[a]UTAUT scale: Score for individual questions 1 (strongly disagree) – 5 (strongly agree). Each domain has 3 questions; domain score ranges from 5-15. Total score for all 6 domains range from 30 – 90

[b]Diff: difference between beginning and end of phase 1 or phase 2

[c]Tot diff: difference between beginning of phase 1 or 2 and phase 3

[d]Mean diff: average of total difference of all participants in each group by domain

[e]Total score: UTAUT score for all 6 domains for individual participants

[f]NA: participant 7 expires prior to phase 3
