# Supplementary material for: Optimization of the Chronic Kidney Disease–Peritoneal Dialysis App to Improve Care for Patients on Peritoneal Dialysis in Northeast Thailand: User-Centered Design Study
Source: JMIR Form Res. 2022 Jul 6;6(7):e37291. doi: 10.2196/37291 (PMC9301552; doi:10.2196/37291)
Supplement: Multimedia Appendix 6 [file formative_v6i7e37291_app6.pdf]

Multimedia Appendix 6: Summary of Participant Observation Phases 1, 2 and 3

Task	Open screens	Enter body weight with NFC	Enter Blood Pressure	Enter Dialysate	View hydration metrics	Interpret hydration metrics	Clinic communication	User incentives
**Phase 1**								
Liked	easy, not complicated	-data automatically entered	-data automatically entered	-data automatically entered	- previous data for BP, UF displayed on the main page	Manual<br>- outlier alert of BW (compared to DW), BP	-easy to contact via LINE and telephone	- data in one place<br>- communicates data to the PD team<br>- manual entry helpful if problem with NFC
Disliked	-app opens slowly<br>-app sometimes unstable	- data not displayed on the weight scale<br>- no alert indicating the device is ready to use<br>- long time to transfer data from card<br>- hard to use hold card in position<br>- inaccurate scales<br>- requires too many steps	- unreadable/ inaccurate if numbers and rows of the BP machine are not the same size or low light<br>- have reading does not match actual value sometimes	- same issues as entering daily body weight<br>- not distinguish between CAPD and APD PD systems	- previous hydration metrics not included<br>- every dialysate volume cycle reported as cycle 1, so UF for each cycle calculated for entire day<br>- slow to load program<br>- no home button to navigate back to the main page<br>- restart required to view previous data<br>- graph does not show hydration, dialysis fluid data<br>- font too small	NFC<br>- no interpretation and alert for BW, BP, dialysate volume, BMI<br>Manual<br>– no interpretation of net dialysate gain/loss<br>- no BMI interpretation	- did not receive an instant reply via LINE from PD clinic	- no feedback or reminders for patients to send data<br>- app is sometimes unstable<br>- can't use app in iOS system
Feature rating[a] mean (range)	**1.07 (1-2)**	**1.07 (1-2)**	**1.07 (1-2)**	**1.14 (1-2)**	**1.29 (1-2)**	**1.07 (1-2)**	**1 (1)**	**1.07 (1-2)**
**Phase 2**								
Liked	easy, automatic login, clear, multifunction, optimal detail	easy, data linked automatically, more detailed value (with a decimal point)	- easy to do<br>- data linked automatically	- easy to do<br>-data linked automatically	historical data for BP, PR, dialysate volume displayed on the main page	Manual<br>- interpretation and alert of BMI, PR and BP<br>- can adjust selfcare	- easy to contact via LINE or telephone or in-person<br>- can consult on various issues	- easy, fast<br>- save data in one place<br>- communicate data to the treatment team
Disliked	-takes a long time to open<br>-need to open multiple pages	- data not displayed on the weight scale<br>- no alert indicating that device is ready<br>- takes a long time for information to transfer while touching the card<br>- too many steps<br>- require multiple devices<br>- inaccurate scales	- unreadable/ inaccurate in low light or if screen has other numbers<br>- must repeat because the numbers do not always match the actual value<br>- no memory in device so need to take photos each time	-same as entering daily body weight<br>- can only edit data<br>-inflow and outflow times not recorded<br>- can't use in APD<br>- cycles after midnight counted in the next day	-no previous data for BW, BMI and fluid intake included<br>- hard to read, not displayed as table<br>- dialysate volume: combined cycles for CAPD<br>- graphical sometimes incomplete (no UF)<br>- graph scale incorrect	NFC<br>- no interpretation and alert of BW, PR, BP, dialysate volume, BMI<br>Manual<br>- no comparison BW to and UF<br>- interpretation not on same page<br>- no alert with sound, different color or font	none	none
Feature rating[a] mean (range)	**1 (1)**	**1.2 (1-2)**	**1.6 (1-3)**	**1.2 (1-2)**	**1 (1)**	**1.2 (1-2)**	**1(1)**	**1.2 (1-2)**
**Phase 3: CKD-PD app**								
Liked	-easy<br>-automatic login	- easy<br>- convenient	- easy to enter<br>- easy to fill out<br>- editable<br>- photo is easy to use<br>- easy to use manual	- easy<br>- automatically calculate<br>- editable	-previous data for BP, PR, dialysate volume displayed on the main page<br>-can see anytime	- interpretation and alert of BW, BMI, BP<br>- know how health is<br>- know what to do next<br>- can adjust selfcare	- easy to contact via LINE or telephone or in-person<br>- a group line<br>-easy to consult clinic	- convenient<br>- easy to carry<br>- data saved together<br>- selfcare planning<br>- faster way to

					- can compare with previous readings<br>-easy to read<br>-can see in one place	- good and instant result update		communicate data to the treatment team<br>- solve problems instantly, may not need to visit hospital
Disliked	-take a long time to open especially body weight icon<br>-app freezes often, must close and reopen it<br>-bounce off by itself	<u>Manual</u><br>-take a long time to open<br><u>NFC</u><br>- take a long time<br>-multiple steps<br>-inaccurate data	- taking photo takes longer than manual entry<br>- difficult to take photo<br>- the numbers are incomplete when taking photo	<u>Manual</u><br>- too many fields<br>- app freezes; must close and reopen<br>- option to adjust time<br>- separate APD & CAPD<br>- not calculating UF<br>- can't delete data<br><u>NFC</u><br>-multiple steps	none	- confusing -BMI is interpreted as being overweight. But in the results of the weight interprets as normal weight	none	-need good internet signal<br>-unstable<br>-app freezes often
Feature rating[a] mean (range)	**1.6 (1-3)**	**<u>Manual</u>**<br>**1.1 (1-2)**<br>**<u>NFC</u>**<br>**2.3 (1-3)**	**1.1 (1-2)**	**<u>Manual</u>**<br>**1.1 (1-2)**<br>**<u>NFC</u>**<br>**2.3 (1-3)**	**1.1 (1-2)**	**1.1 (1-2)**	**1 (1)**	**1.2 (1-3)**
**Phase 3: Logbook**								
Liked	-NA	-easy	- easy<br>- can be inspected and edited	- easy<br>- there are clear fields to fill out	- can see historical data for BP, BW, UF with daily difference<br>-easy to read<br>-doctors can adjust treatment at clinic<br>-can adjust selfcare at home ex. salt restriction, adjust medication	**NA**	- easy to contact via LINE, phone or in-person<br>- exchange opinions<br>- consult on various issues	- easy to see/read<br>- familiar with it<br>- keep as evidence and memory<br>- do not need internet/cellular<br>- can't be hacked<br>- some suggest using both book and app
Disliked	-NA	- inconvenient when logbook not available or paper runs out<br>- small letters<br>- need to do	- inconvenient	- inconvenient<br>- takes a long time<br>- need to calculate UF<br>- tables provided have unnecessary fields<br>- must record the start & stop times beginning & end of each cycle<br>- must separate cycles and days by yourself	- no interpretation<br>- difficult to read because of the large numbers and multiple fields	**NA**	-sometimes difficult to contact via telephone because phone is busy<br>-no private group in LINE<br>-have to wait at clinic visit	-inconvenient to carry<br>-want to stop using the book if there's an app that can replace the book<br>-some feel that it's redundant if using both book and app
Feature rating[a] mean (range)	**NA**	**1.4 (1-2)**	**1.4 (1-3)**	**1.8 (1-3)**	**1.7 (1-3)**	**NA**	**1.2 (1-3)**	**1.9 (1-3)**

*Feature rating scale: 1= Good, 2=Neutral, 3=Not good

Abbreviations:

BP- blood pressure

BW- body weight

DW- dry weight

DW- dry weight

NA – not applicable

NFC – Near Field Communication

UF- ultrafiltration volume
