# Supplementary material for: Optimization of the Chronic Kidney Disease–Peritoneal Dialysis App to Improve Care for Patients on Peritoneal Dialysis in Northeast Thailand: User-Centered Design Study
Source: JMIR Form Res. 2022 Jul 6;6(7):e37291. doi: 10.2196/37291 (PMC9301552; doi:10.2196/37291)
Supplement: Multimedia Appendix 7 [file formative_v6i7e37291_app7.pdf]

Multimedia Appendix 7: Detailed hydration metric completion results

Participant	1	2	3	4	5	6	7[d]	8	9	10
**Phase 1[a]**										
**BW[e] N**	14	11	14	14	12	-	-	-	-	-
**BW done**	12	8	14	7	12					
**BW%**	85.7	72.7	100	50	100	-	-	-	-	-
**BP [f]N**	14	11	14	14	12	-	-	-	-	-
**BP done**	14	10	14	14	12					
**BP %**	100	90.9	100	100	100	-	-	-	-	-
**UF[g] N**	56	44	14	56	24	-	-	-	-	-
**UF done**	54	21	14	52	24					
**UF%**	96.4	47.7	100	92.9	100	-	-	-	-	-
**All HM[h] N**	84	66	42	84	48	-	-	-	-	-
**All HM done**	80	39	42	73	48					
**All HM %**	95.2	59.1	100	86.9	100	-	-	-	-	-
**Phase 2[b]**										
**BW N**	-	-	-	-	-	14	13	14	7	10
**BW done**						13	12	14	0	9
**BW%**	-	-	-	-	-	92.9	92.3	100	0	90
**BP N**	-	-	-	-	-	14	13	14	7	10
**BP done**						14	11	14	0	10
**BP %**	-	-	-	-	-	100	84.6	100	0	100
**UF N**	-	-	-	-	-	14	13	14	28	10
**UF done**						11	12	9	24	8
**UF%**	-	-	-	-	-	85.7	90	76	69.6	76.5
**All HM N**	-	-	-	-	-	42	39	42	30	80
**All HM done**						38	35	37	24	27
**All HM %**	-	-	-	-	-	90.5	89.7	88.1	57.1	90
**Phase 3[c]**										
**BW N**	7	14	14	14	14	14	ND	14	14	14
**BW done**	0	0	3	12	10	14	ND	14	1	11
**BW%**	0	0	21.4	85.7	71.4	100	ND	100	7.1	78.6
**BP N**	7	14	14	14	14	14	ND	14	14	14
**BP done**	7	10	11	11	12	14	ND	14	0	11
**BP %**	100	71.4	78.6	78.6	85.7	100	ND	100	0	78.6
**UF N**	28	56	14	56	28	14	ND	14	56	14
**UF done**	12	32	12	56	18	14	ND	11	14	
**UF%**	42.9	57.1	85.7	100	64.3	100	ND	78.6	25	78.6
**All HM N**	42	84	42	84	56	42	ND	42	84	42
**All HM done**	19	42	26	79	40	42	ND	39	15	33
**All HM %**	45.2	50	61.9	94.0	71.4	100	ND	92.9	17.9	68
**Difference**	-50	-9.1	-38.1	7.1	-28.6	9.5	ND	4.8	-39.2	-11.4

[a] Phase 1: participants 1-5
[b] Phase 2: participants 6-10
[c] Phase 3: participants 1-10
[d]Participant 7 expired before completion of study
[e]BW: body weight
[f]BP: blood pressure
[g]UF: ultrafiltration – volume of fluid removed in 24 hours
[h] HM: Total # of hydration metrics (BW, BP, UF) required for observation period
[f]% of required hydration metrics (BW, BP UF) for observation period completed
