# Supplementary material for: Optimization of the Chronic Kidney Disease–Peritoneal Dialysis App to Improve Care for Patients on Peritoneal Dialysis in Northeast Thailand: User-Centered Design Study
Source: JMIR Form Res. 2022 Jul 6;6(7):e37291. doi: 10.2196/37291 (PMC9301552; doi:10.2196/37291)
Supplement: Multimedia Appendix 8 [file formative_v6i7e37291_app8.pdf]

Multimedia Appendix 8: Issues with Recommended Solutions from App Improvement Team

No.	ISSUE	RECOMMENDATION
Phase 1		
1.1	NFC: Does not have outlet; not enough power	Provide a power plug with a USB connector
1.2	NFC: Scale too small, unstable to stand on. Difficult to bend over to tap card on the machine	Create an extension device with tongs to hold the card so do not have to bend over
1.3	NFC: Lacks data display and alert indicating that the device is ready to use	Instruct user to wait until green light appears, and wait 5 seconds before standing on machine and tapping card
1.4	APP: Different types of data on same graph with the same scale making it hard to read	Displays each type of data on different graphs
1.5	APP: No BMI and dialysate volume interpretation	Add BMI and dialysate volume interpretation
1.6	APP: Can't use in iOS system	Make an app in iOS system
1.7	APP: Display every cycle as cycle 1	Send results as total daily ultrafiltration volume
Phase 2		
2.1	NFC: scale too small and unstable. Cannot stand on it safely. Hard to use extension device to tap card	Make larger and add a handle.
2.2	APP: Scale on graph incorrect, shows different types of data in the same graph	Display each type of data in different graphs
2.3	APP: no comparison between BW and DW; no dialysate volume interpretation	Determine criteria or BW (compare to DW) and dialysate volume interpretation
2.4	APP: no previous data for BW, BMI and fluid intake displayed on the main page	Display previous data for BW, BMI and fluid intake on the main page
2.5	APP: dialysis cycles ending after midnight are counted in the next day	Send results as total daily ultrafiltration volume
2.6	TCC: data not uploading to TCC properly e.g. combined and split CAPD cycles	Send results as total daily ultrafiltration volume
2.7	TCC: No alert on display for PD staff	Activate the alert system in Thai Care Cloud via LINE or SMS
Phase 3		
3.1	APP: Opens slowly, especially BW and UF icons	Improve app so opens faster -Close unused apps
3.2	APP: Freezes often - must close and reopen it	Make the app more stable
3.3	APP: Logs off by itself	Make the app more stable
3.4	APP: Interpretation of BMI and BW confusing	Use Thai so do not confuse with English words for weight and BMI
3.5	APP: Must record height every time for BMI	Automatically enter height recorded during registration
3.6	APP: Not adding dialysate exchange times automatically	Automatically use the time when dialysate has finished draining into the field when dialysate starts to enter as default
3.7	APP: Confusing results of dialysate volume	Suggest using different words that are less confusing for net amount of fluid removed
3.8	APP: No 'Pop-up' to inform that data has been sent or recorded	Add 'Pop-up' to inform that data already sent or recorded
3.9	APP: Can't add in data if forgot	Create way to fill in previous data
3.10	APP: Default time stamp as time of data entry rather than actual time of cycle	Set time of data entry as default, but allow it to be changed to actual time of cycle

APP: CKD-PD app
TCC: Thai Care Cloud
BMI: Body Mass Index
NFC: Near Field Communication
BW: Body weight
DW: Dry weight (after dialysis)
